# Perspectives on hepatitis A and B screening and immunization at a syringe services program: a mixed-methods study

**DOI:** 10.1186/s12954-025-01391-w

**Published:** 2026-01-06

**Authors:** Subul Malik, Marina Plesons, Monica Faraldo, Tyler S. Bartholomew, Hansel E. Tookes, Edward Suarez, David W. Forrest

**Affiliations:** 1 Department of Medicine, University of Miami Miller School of Medicine, Miami, FL USA; 2 Department of Public Health Sciences, University of Miami Miller School of Medicine, Miami, FL USA; 3 Department of Psychiatry and Behavioral Sciences, University of Miami Miller School of Medicine, Miami, FL USA; 4 Department of Anthropology, University of Miami, Miami, FL USA

**Keywords:** Hepatitis A virus, Hepatitis B virus, People who inject drugs, Syringe services programs, Harm reduction

## Abstract

**Background:**

People who inject drugs (PWID) are at increased risk for viral hepatitis, yet hepatitis A virus (HAV) and hepatitis B virus (HBV) screening and immunization rates remain low. Although offering HAV and HBV services at syringe services programs (SSPs) is effective, few U.S. SSPs currently offer them. Limited qualitative research exists on the advantages and optimization of these services at SSPs. This study explored PWID and SSP staff perspectives regarding barriers to HAV and HBV prevention and care services in traditional healthcare, facilitators for SSP-based provision, and opportunities to improve service delivery.

**Methods:**

This study was conducted at an SSP in Miami, Florida serving over 2500 PWID annually. Quantitative data on vaccine administration from August 2023 to May 2025 were abstracted from the SSP database. Prior to implementation, in May 2022, we conducted in-depth interviews with 15 PWID and 11 SSP staff. Transcripts were analyzed using codebook thematic analysis in Dedoose.

**Results:**

From August 2023 to May 2025, the SSP administered 114 HAV and 176 HBV vaccine doses. Qualitative interviews from May 2022 revealed several key findings. Barriers included limited knowledge, stigma and discrimination, resource and transportation challenges, navigation difficulties, and limited prioritization. Facilitators for SSP-based services included the benefits of co-located, on-demand care, and non-stigmatizing and supportive environment. Opportunities for improvement included offering incentives, expanding outreach, and increasing communication.

**Conclusion:**

PWID face significant barriers to HAV and HBV services in traditional healthcare, including stigma, logistical challenges, and limited awareness of viral hepatitis. Integrating these services into SSPs enhanced accessibility and uptake by leveraging trust, convenience, and harm reduction principles.

**Supplementary Information:**

The online version contains supplementary material available at 10.1186/s12954-025-01391-w.

## Introduction

Viral hepatitis remains an important public health challenge, affecting more than 300 million people globally [1]. Despite the availability of safe and effective vaccines, acute hepatitis A virus (HAV) and hepatitis B virus (HBV) infections persist in the United States (US), with 2265 and 2126 new cases reported in 2022, respectively [2]. HAV is primarily transmitted through the fecal-oral route, while HBV is spread through blood or sexual contact [2]. Florida has one of the highest acute HAV and HBV incidence rates in the country, at 1.4 and 3.5 per 100,000, respectively, with significant annual increases [2]. Miami-Dade County currently leads the state in HBV incidence at 4.1 per 100,000 and reported an HAV incidence rate of 1.1 per 100,000 in 2022 [3, 4]. Although hepatitis C virus (HCV) is also a significant public health concern, this study primarily focuses on HAV and HBV given their recent high incidence trends and because they are preventable with vaccines.

People who inject drugs (PWID) are a priority population for viral hepatitis prevention and treatment due to heightened risks associated with homelessness, unsanitary living conditions, and the use of contaminated injection equipment. Injection drug use (IDU) is an identifiable risk factor for new acute HAV and HBV cases, with 299 and 239 national cases identified in 2022 among PWID, respectively [2]. Likewise, 21% of all HAV cases in Florida in 2023 were attributable to IDU [4]. Despite these risks, immunization rates for HAV and HBV remain low among PWID in the US, with only 29% reporting receiving at least one dose of HAV vaccine, HBV vaccine, or both in 2009 [5]. In 2018, 32.4% of PWID in Wisconsin had received at least one dose of the HAV vaccine, and 56.4% had completed the HBV vaccine series [6]. More recently, in 2021, fewer US adults who use drugs in Los Angeles, Atlanta, or Las Vegas reported having received the HAV (45.9%), HBV (47.5%), or influenza vaccines than those for MMR (57.1%) or Td/Tdap (61.1%) [7]. Low vaccine and screening uptake is largely due to the significant barriers PWID face in accessing healthcare services, including lack of health insurance, transportation challenges, stigma, and discrimination, as well as competing priorities, such as housing and basic survival needs [8–10]. Although these data provide useful national and regional context, to our knowledge, no published or publicly accessible data report HAV or HBV vaccination coverage specifically among PWID in Florida. This limitation underscores an important gap in understanding immunization needs among PWID in Florida and highlights the value of establishing mechanisms, including integrating vaccine status into routine surveillance or implementing systematic vaccination tracking within SSPs to support future monitoring and inform targeted vaccination strategies.

The disproportionate burden of viral hepatitis among PWID underscores the need for adjunctive interventions and implementation strategies to engage this population, including expanding the provision of screening and immunization services to syringe services programs (SSP) [7, 11]. While the primary purpose of SSPs is the provision of new injection equipment and disposal of used supplies, they have been increasingly utilized to engage PWID with other healthcare services, given their non-stigmatizing environments and established foundation of trust [12, 13]. Over the past 30 years, studies in the United States have demonstrated the feasibility, effectiveness, and cost-saving benefits of offering HAV and HBV immunization services and screening at SSPs [6, 14–16]. Prior studies aimed at increasing immunization among PWID through SSPs have primarily examined the use of financial incentives and expedited vaccination schedules [17–19]. Recognizing this, the World Health Organization (WHO) recommends that viral hepatitis immunizations be offered at SSPs [11, 20]. Likewise, the Centers for Disease Control and Prevention (CDC) recognizes the critical role that SSPs play in reducing viral hepatitis transmission among PWID [21, 22].

Despite national and international recommendations to integrate HAV and HBV screening and immunization at SSPs, only 30% of SSPs in the United States offered HBV immunizations in 2021 [23]. Meanwhile, data on the number of SSPs that offer HAV immunizations remain limited [23, 24]. Additionally, there is relatively little qualitative research on the perspectives of PWID and SSP staff on the relative advantage of offering these services at SSPs, implementation barriers, and opportunities for service optimization [23–25]. Notably, no studies to date to our knowledge have examined this topic in the Florida context, which is especially relevant given its high HAV and HBV incidence. This study seeks to examine the perspectives of clients and staff at an SSP in Miami, Florida. Specifically, it explores the barriers PWID face when accessing HAV and HBV screening and immunization in traditional healthcare settings, identifies the facilitators that support delivery of these services at SSPs, and investigates strategies to improve SSP-based HAV and HBV screening and immunization to increase uptake.

## Methods

### Setting

This study was conducted at the IDEA Miami SSP, which was established in 2016 as the first legally sanctioned SSP in Florida. As of 2025, it had enrolled over 2500 PWID through its fixed site and mobile unit. In addition to anonymous syringe exchange, it offers an increasingly comprehensive package of harm reduction services, including naloxone training and distribution; wound care; substance use disorder treatment; HIV, Hepatitis C, and sexually transmitted infections (STI) prevention, testing, and treatment; general health maintenance screenings; phlebotomy services; and linkage to social services. Medical services are provided in-person through the confidential arm of the program during the SSP’s two weekly clinics and via telehealth outside clinic hours. It began offering influenza vaccines in November 2020 and expanded to COVID-19 vaccines in December 2021 and HAV and HBV vaccines in August 2023. From August 2023 to May 2025 (the HAV and HBV immunization period discussed in this paper), the anonymous syringe exchange conducted 2489 service encounters with 1065 unique individuals. Although staff encourage anonymous syringe exchange clients to access confidential services at the program’s clinic, many anonymous clients do not agree to access these confidential services resulting in much lower numbers of clients accessing confidential services than those who access the anonymous syringe exchange.

### Vaccine administration protocol

Vaccines at the program’s confidential on-site clinic are offered to anonymous syringe exchange participants on a voluntary, on-demand basis. Efforts to increase immunization rates include flyers and social media posts featuring CDC and Florida Department of Health (FDOH)-approved messages about HAV and HBV, as well as verbal recommendations from SSP staff. Anonymous syringe exchange participants interested in vaccines are directly linked to the program’s confidential clinic and asked about their immunization history, which—with their consent—is verified through Florida SHOTS, the statewide online immunization information system. Blood-based laboratory antibody testing is conducted to determine immunity from HAV and HBV due to prior immunization or past infection. Vaccines are administered by a staff nurse or medical assistant at the SSP fixed site or on its mobile unit. All vaccines are procured from FDOH as part of a formal contract funded by CDC. The vaccines are stored on site in a dedicated refrigerator with temperature monitoring by FDOH. The FDOH contract also provides funding for partial effort for several staff involved in mobile and social media outreach, linkage to confidential HAV and HBV screening and vaccination, and linkage to HCV treatment for this project.

### Data collection

The study was approved by the University of Miami Institutional Review Board and follows the consolidated criteria for reporting qualitative research (COREQ) (Supplementary File 1). In May 2022, we conducted in-depth semi-structured interviews with 15 PWID and 11 staff of the SSP. PWID were eligible for inclusion if they were ≥ 18 years old, had a history of IDU, were enrolled as a participant of the SSP, and were able to consent to participate in the study in English. Staff were eligible for inclusion if they were ≥ 18 years old, worked at the SSP at the time of data collection (May 2022), and were able to consent to participate in the study in English. We approached all participants with information about the study in-person, and they provided informed consent prior to study activities.

Interviews took place in-person in a private location at the SSP (all client interviews) or over Zoom (some staff interviews) and were conducted by two of the co-authors (MF and DF) with training in qualitative methods. The interview guide was structured according to CDC and FDOH directives for this project and approved by the FDOH prior to University of Miami IRB review (Supplementary File 2). Interviews lasted an average of 20 min, and all participants received $25 as compensation. The interviews were audio-recorded and transcribed verbatim in English by a third-party transcription service.

Quantitative data on the number of HAV and HBV vaccines administered to clients at the SSP were abstracted from the SSP’s administrative dataset.

### Data analysis

Interview transcripts were analyzed using codebook thematic analysis, in which themes are determined in advance of analysis and are drawn from established frameworks or theories, existing knowledge of the topic, and/or the interview guide [26]. Using Dedoose (version 9.0.107, Sociocultural Research Consultants, Los Angeles, CA), two study members (SM and MP) reviewed two transcripts and reached consensus on a structured codebook. The same two study members then analyzed all remaining transcripts simultaneously, with minor additional modifications to the codebook. Differences in coding were negotiated until consensus was reached. Once all transcripts were coded, codes were condensed into salient themes for each research question.

Validation strategies included collaboration and member checking [27]. With regard to collaboration, all but one of the co-authors are staff at the SSP. For purposes of participant validation, the themes were presented to the SSP’s staff, and their feedback was incorporated into the analysis.

## Results

### HAV and HBV immunization service provision

From August 2023 to May 2025, a total of 114 and 176 HAV and HBV vaccine doses respectively, were administered at the SSP. For the HBV vaccine series, which was offered as a three-dose series (Engerix-B) during the study period, 83 received 1 dose, 30 received 2 doses, and 11 completed the vaccine series.

### Qualitative themes

Sociodemographic characteristics of the PWID participants and staff who participated in the qualitative interviews are provided in Tables 1 and 2. Most PWID participants were 40 years old or younger (54%) with an approximately equal proportion of men (47%) and women (53%). The majority of PWID identified as White (80%) and non-Hispanic (67%). The SSP staff were relatively more racially diverse and held a variety of positions including case managers, outreach coordinators, research associates, physicians, and leadership.

**Table 1:** Sociodemographic characteristics of PWID participants

Sociodemographic characteristic	*N* (%)
Age (years)	
21–30	4 (27%)
31–40	4 (27%)
> 40	7 (47%)
Sex	
Male	7 (47%)
Female	8 (53%)
Race	
Black	2 (13%)
White	12 (80%)
Other	1 (7%)
Ethnicity	
Hispanic	5 (33%)
Non-Hispanic	10 (67%)

**Table 2:** Sociodemographic characteristics of SSP staff participants

Sociodemographic characteristic	*N* (%)
Age (years)	
21–30	2 (15%)
31–40	10 (77%)
> 40	1 (8%)
Sex	
Male	5 (38%)
Female	8 (62%)
Race	
Black	5 (38%)
White	8 (62%)
Other	0 (0%)
Ethnicity	
Hispanic	5 (38%)
Non-Hispanic	8 (62%)

Qualitative themes were identified for each of the three research questions in Table 3.

**Table 3:** Research questions and qualitative themes

Research Questions	Themes
What are the barriers that PWID experience to accessing HAV and HBV screening and immunization in the traditional healthcare system?	Knowledge and understanding
	Stigma and discrimination
	Resources and transportation
	Navigating the health system
	Relative prioritization
What are the facilitators when offering HAV and HBV screening and immunization at an SSP?	Co-located services
	On-demand services
	Non-stigmatizing and supportive environment
What opportunities exist to optimize SSP-based HAV and HBV screening and immunization services?	Financial and non-financial incentives
	Expanded outreach
	Increased communication

### Barriers to accessing HAV and HBV screening and immunization in the traditional healthcare system

#### Knowledge and understanding

Lack of knowledge about HAV and HBV, including their transmission routes, risk factors, health consequences, and prevention options, was a significant barrier for most SSP clients to accessing HAV and HBV screening and immunization services in the traditional healthcare system. Many SSP clients were unaware of the risks of viral hepatitis or the benefits of vaccination. One reported, *“I wasn’t really informed about it.”* Participants also shed light on vaccine hesitancy, misinformation, and fear of test results as further barriers to seeking screening services. Finally, a few participants noted that their peers have other needs that take priority over knowing their HAV or HBV status.

Staff members echoed similar concerns about this knowledge gap. One emphasized the need for enhanced education efforts: *“Just give them the knowledge to where it actually is somethin’ that could help them…explain to them how it could protect them*,* given them the science behind it.”* Another staff member agreed, adding, *“I think perhaps not understanding the illness…and then the importance of the vaccination.”* Without relevant information, many PWID do not perceive their risk for HAV and HBV or recognize the need for vaccination, leading to low engagement with preventive services.

#### Stigma and discrimination

All clients recounted negative experiences with stigma and discrimination in the traditional healthcare system. One client recalled going *“[to the health department] once…I had got syphilis…they just weren’t very*,* like*,* friendly. I felt very judged.”* Experiences like this left many feeling unwelcome and uncomfortable seeking further care, particularly due to perceived judgment linked to their substance use.

Staff members also recognized stigma as a significant barrier. One remarked, *“Stigma [is a] universal problem*,* unfortunately…why this population may not feel as comfortable seeking out the screenings.”* Another emphasized the compounded impact of stigma tied to both drug use and certain diseases, like HAV and HBV, stating, *“There’s also stigma against certain diseases even on people that are homeless or people that use drugs.”* This layered stigma fostered fear of judgment and discrimination, deterring many PWID from pursuing necessary screening and vaccinations.

#### Resources and transportation

Lack of financial stability and/or health insurance, transportation, and housing were additional barriers to accessing HAV and HBV screening and immunization for many PWID. Travel-related issues, including time and cost, were the most frequently cited barrier. One client explained, *“Traveling time and traveling costs and things like that are really out of the sorts for people who do drugs.”* Another highlighted the difficulty of reaching services without mobile outreach, stating, *“If you guys don’t come around…it’s hard for us to get down here.”* Financial barriers also extended to the real and perceived costs of screening and vaccination services. A staff member noted, many clinics in the traditional healthcare system *“make them pay for it even if they don’t have an income*,* even though they say they’re free.”* These challenges often deterred PWID from seeking preventive care.

#### Navigating the health system

SSP clients faced significant challenges navigating the traditional healthcare system. One major issue was the complexity and delays of scheduling appointments. As one client described, *“It’s such a process*,* and it’s so drawn out*,* and then you don’t know if they’re gonna wanna [involuntarily admit] you…people just don’t like hospitals in general.”*

Staff reiterated these concerns, noting how the rigidity and complexity of the traditional healthcare system make accessing consistent care nearly impossible for many PWID, leading to disengagement. One staff member explained, *“[PWID] have to jump through so many hoops in order to get treatment…multiple follow-up appointments…most of our participants are homeless and honestly just cannot keep track of when they have to come in.”* Missed appointments due to lack of technology, unpredictable living conditions, and lack of IDs often resulted in lost access to care, compounding the problem. Additionally, the system’s slow pace discouraged engagement. As another staff member noted, *“They’ll walk out on the appointments…It has to be very quick for them to really engage.”*

#### Relative priority

For many SSP clients, meeting immediate survival needs—such as securing income, accessing substances, obtaining food, and finding safe shelter—often took precedence over preventive healthcare, including HAV and HBV screening and vaccination. One client captured the urgency of substance use, saying, *“If you’re sick from dope shit*,* you want that dope in you…until then you don’t want to be messing around with stuff.”* Another client noted that prioritization is highly individual, stating, *“It all depends on the person’s desire…whether it’s the addiction issue*,* whether it’s treatment or…immunization…It’s*,* basically*,* up to the person.”*

Despite these barriers, staff observed that participants are generally open to viral hepatitis prevention and treatment when it is accessible, with one staff member noting, *“Every participant I came across is open for treatment. They do wanna be treated.”* However, the competing demands of survival make it difficult for many to prioritize preventive care, highlighting the importance of offering flexible, low-barrier services that can better meet the needs of this population.

### Facilitators that support the delivery of HAV and HBV screening and immunization at an SSP

#### Co-located services

Co-located services emerged as a crucial facilitator for promoting HAV and HBV screening and immunization among PWID at the SSP. Clients and staff both highlighted the benefits of having multiple services that PWID need in one location. One client described their appreciation for this convenience, saying, *“They’re a one-stop-shop. You can go to them. You can see a doctor. You can get enrolled fast. Everything’s on site there.”* This streamlined access encouraged clients to engage with services they might otherwise delay or avoid. A staff member observed, *“They might be coming in for one thing*,* but you offer this*,* this*,* and this here. You can have all that done today.”* Another staff member emphasized the advantage of a fully integrated service model, noting, *“That’s what*,* I think*,* would be all of our dreams…they can get their immunization shots here…do everything here.”*

#### On-demand services

The SSP’s model of on-demand services was identified as a second key facilitator for HAV and HBV screening and immunization. Both clients and staff emphasized the importance of receiving care immediately, without the delays often experienced in traditional healthcare settings. As one client emphasized their preference for receiving services, *“On the spot*,* when you need them*,* at the time you need ’em.”* Staff also recognized these benefits, with one noting, *“Whoever walks in for their enrollment or quarterly [assessment]*,* they can just get immunized right then and there for Hep A and B.”* This model eliminates the need for clients to wait for or schedule separate appointments. Another staff member highlighted the significance of this: *“We only have a certain window to be able to give people these immunizations*,* but I think having it available at a SSP is vital for people who inject drugs.”*

#### Non-stigmatizing and supportive environment

The SSP’s non-stigmatizing and supportive environment, rooted in a harm reduction approach, was a third facilitator for HAV and HBV screening and immunization. Clients appreciated the respectful and friendly atmosphere. One client emphasized, *“[The SSP staff] are really nice*,* and they’re not judgmental. I really would be upset if they went away.”* This welcoming environment helped build trust, making clients more willing to engage in care. Staff also recognized the importance of this approach, with one noting, *“What makes it easier is just having staff who practice non-judgmental stances and having staff engage these individuals who understand the lifestyle and the options.”* This approach fosters rapport and encourages open communication about health concerns. Another staff member added, *“Providing compassion*,* meeting them where they’re at*,* and providing evidence-based care. I think that’s important.”* The harm reduction model, grounded in meeting clients where they are, removed the fear of judgment among PWID, creating a space where clients feel comfortable seeking care, including HAV and HBV screening and immunizations.

### Opportunities for further SSP-based HAV and HBV screening and immunization improvements

#### Financial and non-financial incentives

Both clients and staff suggested that offering both financial and non-financial incentives could significantly increase engagement with HAV and HBV screening and immunization services at the SSP. One client noted, *“Compensation*,* that’s always a plus out here*,*”* while another added, *“It would be easier if there were money involved. That would bring them out.”* Staff also recognized the value of practical inducements, such as water, snacks, or transportation assistance. One staff member explained, *“I think we should offer them water*,* snacks*,* a bus pass*,* maybe a gift card or something like that. That could help. I think that could go a long way.”* These incentives would address immediate needs, reducing the pressure clients often felt to prioritize substance use or other survival-related activities.

#### Expanded outreach

Expanding outreach services through the SSP’s mobile unit was highlighted as another opportunity to enhance access to HAV and HBV screening and immunization for PWID. Clients underscored the value of *“coming out to the community and*,* basically*,* giving easier access to the system.”* Transportation barriers were a common concern, as one client explained: *“Not everyone has the same options for transportation as the next person.”* Mobile services could address this disparity in access to care by reaching individuals who may otherwise face difficulty accessing the SSP’s fixed site. Staff also recognized the benefits of outreach, with one staff member stating, *“It would be very helpful because we have a lotta people who don’t wanna come to the fixed site.”*

#### Increased communication

Lastly, increased communication and education about HAV and HBV was identified as a third opportunity to improve engagement with screening and immunization services at the SSP. Clients emphasized the need for clear, impactful messaging, such as providing statistics on the prevalence of these infections among PWID. One client suggested, *“Show all the people here the statistics on who all and how many drug users have that*,* and they could’ve gotten outta that by doing what [the SSP] offers.”* Staff echoed the importance of offering comprehensive education, with one recommending, *“Providing individuals or people who inject drugs with access to education on the diagnosis like Hep A and Hep B*,* what it means to live with these conditions*,* and the available treatments.”* Additionally, the role of peer service navigators in facilitating communication was underscored, with one staff member noting, *“It has to do with…the communication skill or the persuasiveness of the person who’s speaking to them.”*

## Discussion

This study explored perspectives from PWID and SSP staff on the barriers PWID encounter accessing HAV and HBV screening and immunization services in the traditional healthcare system, as well as facilitators and opportunities for delivering them at an SSP, especially in a city and state with increasingly high prevalence of viral hepatitis. While prior research has highlighted this significant public health concern globally, there has been limited recent qualitative research exploring how the challenges of HAV and HBV vaccine uptake and screening among PWID manifest, especially in Florida, a high incidence state. Additionally, despite both national and international recommendations to integrate these services into SSPs, data on recent HAV and HBV vaccine availability, especially in high prevalent states, remains limited [7, 23]. This study addresses these gaps by exploring the feasibility, challenges, and opportunities for optimizing these efforts at an SSP.

Barriers to accessing HAV and HBV services in the traditional healthcare system included limited knowledge and understanding of viral hepatitis, stigma and discrimination, resource limitations and transportation challenges, difficulties navigating the healthcare system, and competing priorities. This study aligned with previous research demonstrating that lack of access to vaccines, discomfort with healthcare providers, and dissatisfaction with access to healthcare were prevalent among PWID when surveyed on attitudes towards HAV and HBV [28–32]. It adds nuance to the literature by demonstrating how HAV and HBV may not be a priority for PWID and how limited education with traditional healthcare providers about viral hepatitis further discourages this population from seeking services.

Facilitators to delivering HAV and HBV services at the SSP included the benefits of co-located and on-demand services, and the SSP’s non-stigmatizing and supportive environment based on a harm reduction approach. Integrating HAV and HBV screening and immunization services at locations that PWID frequent—such as SSPs, drug treatment centers, and similar health services—has been shown to increase uptake [12, 33]. For example, the Positive Health Project, an SSP based in New York City, demonstrated that offering on-site HBV vaccinations to PWID resulted in an 83% completion rate of the series [17]. Furthermore, since this population tends to have a high prevalence of comorbidities, integrating various health services at SSPs can improve access to and engagement with broader preventative and curative health services for PWID [6, 13]. This study provides practical examples of how co-located and on-demand services can be effectively implemented. One example of the latter at the IDEA Miami SSP could be adding HAV and HBV immunization status to the intake process during routine SSP visits and quarterly assessments, as patients are already asked about HCV and HIV status at these times. The findings of this study strengthen the case for scaling such interventions to address the unmet needs of PWID.

Opportunities for further SSP-based HAV and HBV service improvements included offering financial and non-financial incentives, expanding outreach efforts, and increasing communication about the viruses. Much existing literature on increasing immunization among PWID through delivery at SSPs has focused on financial incentives and accelerated vaccine schedules [15]. Early studies in Alaska, Connecticut, and New York identified modest financial incentives and convenient location as SSP factors and injecting daily and currently experiencing homelessness as demographic factors that increased vaccine series completion [17–19]. These findings align with WHO’s 2012 recommendation to use incentives to bolster HBV vaccination rates among PWID [20]. Another randomized trial at SSPs in Connecticut and Illinois found that an accelerated vaccination schedule (0, 1, and 2 months vs. 0, 1, and 6 months) was significantly associated with vaccine series completion, but unknown immunity [34]. More recently, new vaccine formulations such as third-generation hepatitis B recombinant vaccines offer the possibility of simplified schedules, such as a 0, 1-month schedule instead of the traditional 0, 1, 6-month schedule for the older HBV vaccine [35]. In clinical trials, two doses of Heplisav-B were more immunogenic than three doses of an older hepatitis B virus vaccine (Engerix-B) [36]. Beginning in June 2025, the IDEA Miami SSP in Miami began transitioning to the Heplisav-B two-dose vaccine in an effort to improve completion rates for its clients. The current protocol is to vaccinate clients prior to checking previous immunity. If the client is immune after the first dose, they do not receive the second dose. This modified protocol can potentially reduce the barriers to services with the time delay between laboratory assessment of titers and vaccine administration.

Additionally, this study reiterates the opportunity of expanded outreach to improve uptake of HAV and HBV screening and immunization services among PWID [25]. In response to these findings, the IDEA Miami SSP now offers these services on its mobile unit, bringing on-demand services directly to the community and reducing resource barriers discussed previously. Likewise, this study offers perspectives on how effective marketing through social media that incorporates both approved messaging and information that resonates with PWID could improve vaccination campaigns. Leveraging trusted messengers, such as peer service navigators, could enhance the effectiveness of educational efforts, helping clients feel more comfortable, informed, and empowered to make decisions about their health.

This study had several strengths and limitations. In terms of limitations, the study was conducted at a single SSP in one city in Florida. Given the unique demographics of Miami-Dade County, the perspectives and findings from the study may not be generalizable to SSPs in other locations. Additionally, our sample was predominantly White and non-Hispanic, limiting its representativeness of the diverse population of Miami-Dade County. Furthermore, although the qualitative interview findings were reported anonymously, the inherent nature of the study may introduce self-reporting bias. Finally, the interviews were conducted prior to the inception of HAV and HBV vaccine implementation at the SSP site. Future studies could investigate optimization strategies to increase the reach of vaccines at the SSP, as implementation of these services progresses. In terms of strengths, this study was conducted in a city with high prevalences of HAV and HBV, and the findings may assist other SSPs in similar contexts. Additionally, few qualitative studies have included perspectives from both SSP clients and staff [23–25]. Finally, this study investigated both HAV and HBV screening and immunization among PWID, whereas prior research has primarily focused on HBV.

## Conclusion

PWID remain a critical population for targeted viral hepatitis prevention and treatment efforts given their increased risk. Delivering HAV and HBV services at SSPs represents a critical strategy to increase reach and accessibility of services, and therefore advance health equity among PWID.

## Supplementary Information

Below is the link to the electronic supplementary material.

Supplementary Material 1

Supplementary Material 2

## Data Availability

Our research data includes sensitive or confidential information and is therefore not publicly available, but are available from the corresponding author on reasonable request.

## Abbreviations

HAV: Hepatitis A virus
HBV: Hepatitis B virus
PWID: People who inject drugs
IDU: Injection drug use
SSP: Syringe services programs
FDOH: Florida Department of Health
