# Supplementary material for: Perspectives on hepatitis A and B screening and immunization at a syringe services program: a mixed-methods study
Source: Harm Reduct J. 2026 Jan 6;23:23. doi: 10.1186/s12954-025-01391-w (PMC12870262; doi:10.1186/s12954-025-01391-w)
Supplement: Supplementary file 1 — Supplementary Material 1 [file 12954_2025_1391_MOESM1_ESM.docx]

**Supplementary File 1: Consolidated criteria for reporting qualitative research (COREQ)**

| **No.** | **Item** | **Guide questions/description** | **Page #** |
| --- | --- | --- | --- |
| **Domain 1: Research team and reflexivity** | | | |
| **Personal Characteristics** | | | |
| 1 | Interviewer/facilitator | Which author/s conducted the interview or focus group? | 5 |
| 2 | Credentials | What were the researcher’s credentials? E.g. PhD, MD | 1 |
| 3 | Occupation | What was their occupation at the time of the study? | 5 |
| 4 | Gender | What was the gender of the researcher(s)? | 1 |
| 5 | Experience and training | What experience or training did the researcher have? | 5 |
| **Relationship with Participants** | | | |
| 6 | Relationship established | Was a relationship established prior to study commencement? | 4-5 |
| 7 | Participant knowledge of the interviewer | What did the participants know about the researcher? e.g. personal goals, reasons for doing the research | 4-5 |
| 8 | Interviewer characteristics | What characteristics were reported about the interviewer/facilitator? e.g. Bias, assumptions, reasons and interests in the research topic | 4-5 |
| **Domain 2: Study Design** | | | |
| **Theoretical Framework** | | | |
| 9 | Methodological orientation and theory | What methodological orientation was stated to underpin the study? e.g. grounded theory, discourse analysis, ethnography, phenomenology, content analysis | 5 |
| **Participant Selection** | | | |
| 10 | Sampling | How were participants selected? e.g. purposive, convenience, consecutive, snowball | 4-5 |
| 11 | Method of approach | How were participants approached? e.g. face-to-face, telephone, mail, email | 4-5 |
| 12 | Sample size | How many participants were in the study? | 4 |
| 13 | Non-participation | How many people refused to participate or dropped out? Reasons? | 4-5 |
| **Setting** | | | |
| 14 | Setting of data collection | Where was the data collected? e.g. home, clinic, workplace | 5 |
| 15 | Presence of non-participants | Was anyone else present besides the participants and researchers? | 5 |
| 16 | Description of sample | What are the important characteristics of the sample? e.g. demographic data, date | 5-6 |
| **Data Collection** | | | |
| 17 | Interview guide | Were questions, prompts, guides provided by the authors? Was it pilot tested? | 5 |
| 18 | Repeat interviews | Were repeat interviews carried out? If yes, how many? | 5 |
| 19 | Audio/visual recording | Did the research use audio or visual recording to collect the data? | 5 |
| 20 | Field notes | Were field notes made during and/or after the interview or focus group? | 5 |
| 21 | Duration | What was the duration of the interviews or focus group? | 5 |
| 22 | Data saturation | Was data saturation discussed? | 5 |
| 23 | Transcripts returned | Were transcripts returned to participants for comment and/or correction? | 5 |
| **Domain 3: Analysis and Findings** | | | |
| **Data Analysis** | | | |
| 24 | Number of data coders | How many data coders coded the data? | 5 |
| 25 | Description of coding tree | Did authors provide a description of the coding tree? | 5 |
| 26 | Derivation of themes | Were themes identified in advance or derived from the data? | 5 |
| 27 | Software | What software, if applicable, was used to manage the data? | 5 |
| 28 | Participant checking | Did participants provide feedback on the findings? | 5 |
| **Reporting** | | | |
| 29 | Quotations presented | Were participant quotations presented to illustrate the themes / findings? Was each quotation identified? e.g. participant number | 7-12 |
| 30 | Data and findings consistent | Was there consistency between the data presented and the findings? | 7-12 |
| 31 | Clarity of major themes | Were major themes clearly presented in the findings? | 6-12 |
| 32 | Clarity of minor themes | Is there a description of diverse cases or discussion of minor themes? | 6-12 |
