# Supplementary material for: Perspectives on hepatitis A and B screening and immunization at a syringe services program: a mixed-methods study
Source: Harm Reduct J. 2026 Jan 6;23:23. doi: 10.1186/s12954-025-01391-w (PMC12870262; doi:10.1186/s12954-025-01391-w)
Supplement: Supplementary file 2 — Supplementary Material 2 [file 12954_2025_1391_MOESM2_ESM.docx]

**Qualitative Interview Guide**

**Identify facilitators and barriers to access of infectious disease screening (specifically Hep A, B, C) by PWID:**

*What makes it easier for PWID to access infectious disease screening?*

*What makes it harder for PWID to access infectious disease screening?*

**Identify facilitators and barriers to infectious disease treatment (specifically Hep A, B, C) by PWID:**

*What makes it easier for PWID to access infectious disease treatment?*

*What makes it harder for PWID to access infectious disease treatment?*

**Identify individual and systems level opportunities to improve service delivery for infectious disease screening and treatment (specifically Hep A, B, C) by PWID:**

*What individual and systems level opportunities are there to improve service delivery for infectious disease screening by PWID?*

*What individual and systems level opportunities are there to improve service delivery for infectious disease treatment by PWID?*
